# The Efficacy, Safety and Satisfaction Associated with Switching from Brinzolamide 1% and Brimonidine 0.1% to a Fixed Combination of Brinzolamide 1% and Brimonidine 0.1% in Glaucoma Patients

**DOI:** 10.3390/jcm10225228

**Published:** 2021-11-10

**Authors:** Hiromitsu Onoe, Kazuyuki Hirooka, Mikio Nagayama, Atsushi Hirota, Hideki Mochizuki, Takeshi Sagara, Katsuyoshi Suzuki, Hideaki Okumichi, Yoshiaki Kiuchi

**Affiliations:** 1 Department of Ophthalmology and Visual Science, Hiroshima University, Hiroshima 734-8551, Japan; onoehir@hiroshima-u.ac.jp (H.O.); oumic@hiroshima-u.ac.jp (H.O.); ykiuchi@hiroshima-u.ac.jp (Y.K.); 2 Nagayama Eye Clinic, Okayama 714-0086, Japan; mikio@po.harenet.ne.jp; 3 Hirota Eye Clinic, Yamaguchi 745-0017, Japan; hirotaganka@lion.ocn.ne.jp; 4 Kusatsu Eye Clinic, Hiroshima 733-0861, Japan; mochizuki-h@hiroshima-u.ac.jp; 5 Sagara Eye Clinic, Yamaguchi 758-0021, Japan; tsagara@sagara-eye.jp; 6 Suzuki Eye Clinic, Yamaguchi 755-0155, Japan; suzuki_eye@grace.ocn.ne.jp

**Keywords:** glaucoma, brinzolamide, brimonidine, satisfaction

## Abstract

We evaluated glaucoma patients for the efficacy, safety and satisfaction associated with switching from brinzolamide 1% and brimonidine 0.1% to a fixed combination of brinzolamide 1% and brimonidine 0.1%. A total of 22 glaucoma patients were enrolled and completed this prospective, nonrandomized study that evaluated patients who underwent treatment with at least brinzolamide 1% and brimonidine 0.1%. Patients on brinzolamide 1% and brimonidine 0.1% were switched to a brinzolamide/brimonidine fixed-combination ophthalmic suspension (BBFC). Evaluations of intraocular pressure (IOP), superficial punctate keratopathy (SPK) and conjunctival hyperemia were conducted at baseline and at 4 and 12 weeks. The Treatment Satisfaction Questionnaire for Medication-9 (TSQM-9) was utilized to assess the change in treatment satisfaction. At baseline and at 4 and 12 weeks, the IOP was 15.0 ± 4.1, 14.8 ± 4.1 and 14.8 ± 4.1 mmHg, respectively. There were no significant differences observed at any of the time points. However, the SPK score significantly decreased at 12 weeks, even though no significant differences were observed for the conjunctival hyperemia incidence at any of the time points. After switching from brinzolamide 1% and brimonidine 0.1% to BBFC, there was a significant increase in the TSQM-9 score for convenience and global satisfaction. Both an improvement in the degree of SPK and an increase in treatment satisfaction occurred after switching from brinzolamide 1% and brimonidine 0.1% to BBFC, even though there were sustained IOP values throughout the 12-week evaluation period.

## 1. Introduction

The degeneration of retinal ganglion cells that results in visual field loss can potentially lead to blindness, and is the primary characteristic of the progressive optic neuropathy that is referred to as glaucoma [1]. The key risk factor for the development and progression of glaucomatous neuropathy is an elevated intraocular pressure (IOP) [2]. Methods for reducing the glaucoma progression risk have been reported by several studies and have all been associated with a lowering of the IOP [3,4].

In order to achieve successful glaucoma management, the use of combination therapy with multiple IOP-lowering medications is suggested when monotherapy proves to be insufficient [5]. Although lowering of the IOP can be achieved through the use of multiple medications, decreased patient adherence to treatment and persistence with the therapy can limit the effectiveness of the treatment [6,7]. However, when fixed combinations are utilized so that a single formulation contains two or more medications, this helps to reduce the dosing frequency in addition to reducing the exposure of patients to preservatives. Therefore, this suggests that improvements in both patient comfort and the adherence to and persistence of the treatment could potentially be achieved when using fixed-dose combinations [8,9].

At the present time, the only fixed-combination glaucoma therapy that does not contain a β-blocker is the brinzolamide/brimonidine fixed-combination ophthalmic suspension. Several studies have reported that the administration of BBFC two to three times daily in open-angle glaucoma (OAG) or patients with ocular hypertension that were adequately controlled with prostaglandin analogs led to an effective lowering of the IOP [10,11,12]. In Japan, the administration of BBFC two times daily was approved for use in 2019.

The current study aimed to evaluate the switching of patients from brinzolamide 1% and brimonidine 0.1% to BBFC, and then investigate the efficacy, safety and satisfaction associated with this change. After the switch in these medications, this meant that patients only needed to use one bottle of eye drops with an instillation of two drops per day, which was much simpler as compared to their previous procedures.

## 2. Materials and Methods

From October 2020 to August 2021, six investigational sites participated in this clinical trial. These sites included Hiroshima University Hospital (Hiroshima, Japan), Nagayama Eye Clinic (Okayama, Japan), Hirota Eye Clinic (Yamaguchi, Japan), Sagara Eye Clinic (Yamaguchi, Japan), Kusatsu Eye Clinic (Hiroshima, Japan) and Suzuki Eye Clinic (Yamaguchi, Japan). The Institutional Review Board of Hiroshima University approved the study protocol. All subjects provided written informed consent for participation in the research study in accordance with the principles outlined in the Declaration of Helsinki.

All patients underwent examinations of visual acuity, refraction, visual field, slit-lamp examination and gonioscopy. Eligibility criteria included an age ≥ 20 years, glaucomatous optic disc abnormalities and corresponding glaucomatous visual field defects, in addition to being treated with at least brinzolamide 1% (Alcon Laboratories Inc, Ft Worth, TX, USA; preservative: 0.005% sodium chloride) and brimonidine 0.1% (Senju Pharmaceutical Co. Ltd., Osaka, Japan; preservative: 0.01% benzalkonium chloride). Exclusion criteria included subjects having active ocular diseases in either eye with the exception of glaucoma, having retinal disease with a potential risk of progression, having undergone ocular surgery or laser treatment within the previous 1 year, being on a regimen for systemic or local administration of steroids during this study or having corneal disease in either eye that potentially posed a problem for accurate IOP measurements. If both eyes of a patient met the inclusion criteria, the analysis used the eye with the higher IOP. When the IOP was the same in both eyes, the analysis used the right eye.

Patient examinations during the study consisted of 3 scheduled visits that occurred over 12 weeks (day 0 and weeks 4 and 12). At day 0 (baseline), patients deemed eligible for enrollment and who were using brinzolamide 1% and brimonidine 0.1% were switched to BBFC (Senju Pharmaceutical Co. Ltd., Osaka, Japan; preservative: 0.003% benzalkonium chloride).

At each visit, patients underwent measurements for IOP, best-correlated visual acuity and biomicroscopic examinations. Using a Goldmann applanation tonometer, with the same procedure performed at all centers, the IOP was measured during the same time as the brinzolamide 1% and brimonidine 0.1% administration. The primary efficacy outcome was defined as the main change in the IOP at 12 weeks from the baseline. Prior to switching to BBFC, the average baseline IOP was determined twice before implementing any changes.

For biomicroscopy, all patients underwent a slit-lamp examination. To assess the conjunctival hyperemia, we used a 4-point hyperemia grading scale that utilized four different photographs to determine the hyperemia matching. These included: 0 = no hyperemia, 1 = slight hyperemia, 2 = moderate hyperemia and 3 = severe hyperemia. Recording of the corneal epithelial disorders during the slit-lamp examination used an A (area) D (density) grading scale [13].

The Treatment Satisfaction Questionnaire for Medication-9 (TSQM-9) was used to assess the change in treatment statisfaction [14]. The TSQM-9 uses a 5- or 7-point scale to determine the assessment, with an increase in the scores indicating there is an increase in the treatment satisfaction. These results can be further grouped in order to indicate the effectiveness, convenience and global satisfaction scores. For these three evaluated areas, the total scores can range from 0 to 100. Greater satisfaction is equated with a higher total score. All patients completed the questionnaires at baseline and again at 12 weeks.

For continuous variables, the Anderson–Darling test was used to assess the variance equality. Based on the results obtained, differences between the baseline and follow-up visits were then assessed by either a Student’s *t*-test or Wilcoxon signed-rank test. Scores for the TSQM-9 are presented as the median and interquartile ranges. All statistical analyses were conducted using JMP software version 15 (SAS Inc., Cary, NC, USA). Statistical significance was indicated by a *P* value of 0.05 or less. All data are presented as the mean ± standard deviation.

## 3. Results

This study evaluated a total of 22 patients (22 eyes), with all of the patients completing 12 weeks of follow-up. Baseline patient demographic data are shown in Table 1. The mean age was 67.2 ± 17.2 years.

**Figure 1:**
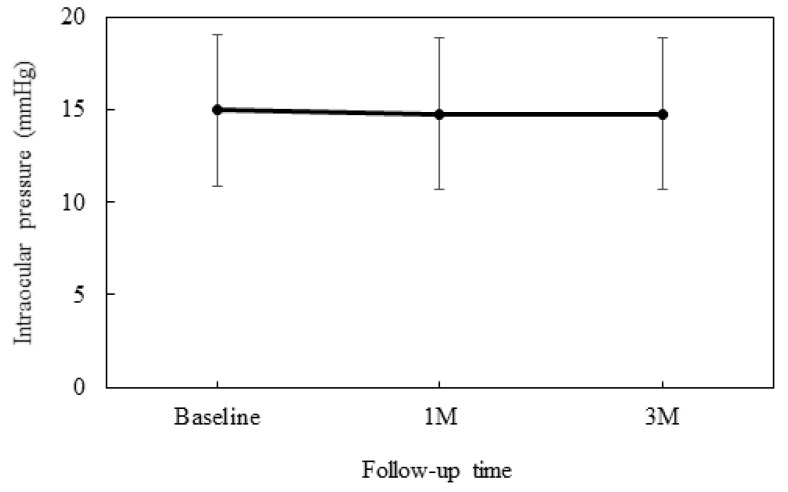
Mean intraocular pressure at baseline and at 4 and 12 weeks. There was no significant difference observed in the mean IOP as compared to the baseline.

At baseline, the IOP was 15.0 ± 4.1 mmHg, while at 4 weeks it was 14.8 ± 4.1 mmHg and 14.8 ± 4.1 mmHg at 12 weeks (Figure 1). No significant differences were observed between the baseline and each of the follow-up visits. Table 2 presents the degree of SPK that was assessed when using the AD grading scale along with the conjunctival hyperemia scores. Although there was no difference between baseline and the 12-week values for the conjunctival hyperemia score, significant improvement was noted for the degree of SPK at 12 weeks.

Comparison of the baseline TSQM-9 scores indicated that patients were satisfied with both the treatment and its convenience. However, there was no significant difference found with regard to the effectiveness (Table 3).

## 4. Discussion

Throughout the 12-week observation period, the IOP values and conjunctival hyperemia were sustained after switching to BBFC from brinzolamide 1% and brimonidine 0.1%. However, there was a significant improvement in the superficial punctate keratopathy and global satisfaction.

Gandolfi et al. [15] examined patients with OAG or ocular hypertension and reported that BBFC as compared to the concomitant administration of an unfixed combination of brinzolamide 1% and brimonidine 0.2% exhibited an IOP-lowering efficacy. When compared to baseline values, the diurnal IOP change at 3 months was −8.5 ± 0.16 mmHg for patients administered BBFC, while it was −8.3 ± 0.16 mmHg for patients administered brinzolamide and brimonidine. Moreover, a similar IOP-lowering efficacy was found for BBFC as compared to that for brinzolamide and brimonidine. While in Japan a 0.1% concentration of brimonidine is currently used, European countries and North America use 0.2% solutions. Brimonidine concentrations used in Japan were determined based on the results of a dose–response trial that evaluated the IOP-lowering efficacy for Japanese subjects. Therefore, when BBFC is administered in Japan, the formulation uses 0.1% brimonidine. During the 12-week observation period in our current study, a sustained IOP was observed after switching to BBFC from brinzolamide and brimonidine.

Both the ocular surface and tear balance are known to be affected by IOP-lowering medications [16]. Furthermore, it should be noted that when using 0.1% brimonidine, it contains 0.01% benzalkonium chloride (BAC) as the preservative, while BBFC contains 0.003% BAC as the preservative. A previous study reported that BAC concentrations higher than 0.01% were associated with toxicity on human corneal epithelial cell sheets [17]. In contrast, 1% brinzolamide does not contain BAC, with 0.005% sodium chloride (NaClO_2_) used as the preservative. A further study that examined corneal epithelial cells following a 30-min exposure to 0.002–0.1% NaClO_2_ reported finding a high survival rate of 80% or greater [18]. Therefore, these results suggest that NaClO_2_ causes relatively little corneal damage. Thus, due to the difference in the BAC concentrations between BBFC and 0.1% brimonidine, there was a significant decrease in the AD grading scale observed after switching to BBFC.

When assessing patients’ satisfaction with treatments, TSQM-9 has proven to be a reliable instrument, with this questionnaire used for treatment satisfaction assessments in various diseases [14,19,20]. Based on these findings, our current study also used the TSQM-9 to assess patient satisfaction. Another benefit of switching to BBFC from brinzolamide and brimonidine is that there was a reduction in both the number of bottles and instillations. After this switch, the previous two bottles of eye drops and instillation of four drops per day were reduced to one bottle of eye drops and an instillation of only two drops per day. Therefore, the results of our current study demonstrated there was an increase in convenience from the baseline 67 to 72 along with a global satisfaction increase from 71 to 79. Other previous studies have also found that there was a low persistence associated with glaucoma medications when patients were required to use multiple medications [7,21,22]. These results suggest that fixed-combination therapies, including BBFC, can potentially alleviate these types of issues.

There were some limitations for the current study. First, only a relatively small number of cases were evaluated in this study. Therefore, multi-center and larger scale trials will need to be undertaken in order to obtain more rigorous, comparative and definitive evidence and data. One other limitation of our current study was that the observation period used was not long enough. Thus, more clear and definitive results could potentially be obtained when using a longer observation period.

In conclusion, both a sustained IOP along with a significant improvement in the degree of SPK were obtained when switching to BBFC from brinzolamide 1% and brimonidine 0.1%. Furthermore, after switching to BBFC, patients who required multiple IOP-lowering medications reported an improved satisfaction with their medical treatment.

## Figures and Tables

**Table 1:** Baseline patient characteristics.

Age (years)	67.2 ± 17.2
Gender (M/F)	12/10
Untreated IOP (mmHg)	20.9 ± 6.0
Baseline IOP (mmHg)	15.0 ± 4.1
Mean deviation (dB)	−10.6 ± 10.0
Type of glaucoma	
POAG	14
NTG	5
Exfoliation glaucoma	3
IOP-lowering medications	
(except brimonidine and brinzolamide)	
PGA	15
PGA/β-blocker	6
None	1

M: male; F: female; IOP: intraocular pressure; POAG: primary open-angle glaucoma; NTG: normal-tension glaucoma; PGA: prostaglandin analogue.

**Table 2:** SPK and hyperemia score.

	SPK Score	*p* Value *	Hyperemia Score	*p* Value *
Baseline	1.18 ± 1.26		0.43 ± 0.75	
1M	0.83 ± 1.10	0.40	0.61 ± 0.85	0.33
3M	0.55 ± 0.92	0.02	0.48 ± 0.75	0.58

SPK; superficial punctate keratopathy; * Calculated using Wilcoxon signed-rank test.

**Table 3:** TSQM-9 scores.

	Baseline (IQR)	3M (IQR)	*p* Value *
Effectiveness	61 (49–68)	67 (50–72)	0.10
Convenience	67 (50–79)	72 (67–85)	0.01
Global satisfaction	71 (62–79)	79 (69–93)	0.02

IQR: interquartile range. * Calculated using Wilcoxon signed-rank test.

## Data Availability

The data analyzed in this study are available from the corresponding author on reasonable request.
